# The benefits of involving general practitioners in the promotion of e-health tools for primary prevention of suicide in the general population: The stopblues case

**DOI:** 10.1192/j.eurpsy.2021.316

**Published:** 2021-08-13

**Authors:** A. Le Jeannic, K. Turmaine, K. Chevreul

**Affiliations:** 1 Urc Eco, Assistance Publique - Hôpitaux de Paris, Paris, France; 2 Eceve Umr1123, Inserm l Université Paris Diderot - Paris 7, Paris, France; 3 Umr1123 -eceve, INSERM, Paris, France

**Keywords:** General Practitioners, Suicide, Primary prevention, e-health

## Abstract

**Introduction:**

In France about 10,000 suicides/year are recorded. General practitioners (GPs) have an important role in prevention, with consultation rates between 20% and 76% the day preceding suicide. StopBlues is an application/website for primary prevention of suicide in the general population. Its promotion was supported by municipalities and involved GPs.

**Objectives:**

To evaluate how the involvement of GPs in the promotion of StopBlues had an impact on its utilization.

**Methods:**

StopBlues was promoted in 25 French municipalities randomly assigned to a ‘basic’ promotion group organized by municipalities only or an ‘intensified’ promotion group that also includes promotion in GPs’ waiting rooms. StopBlues users were asked how they found out about StopBlues. After two years, an ad hoc questionnaire was sent to all GPs (N=2,111).

**Results:**

StopBlues users from those municipalities (N=885) were 16% to learn about StopBlues from GPs, 93% of them living in municipalities with ‘intensified’ promotion. In the ‘basic’ group, where no GPs have heard about StopBlues, 15% would like to know more about it/will have a look at it and 8% will use it and recommend it to colleagues. Half of GPs from the ‘intensified’ group had heard about the program, with 24% who recommended StopBlues to some patients. 21% of GPs agreed that they will use it and recommend it to colleagues.

**Conclusions:**

Involving GPs in the use of e-health tools is of major interest to improve their utilization. Our results show that GPs are in need of those in dealing with patients with psychological pain/distress.

**Disclosure:**

No significant relationships.

